# Gender-Specific Cytokine Pathways, Targets, and Biomarkers for the Switch from Health to Adenoma and Colorectal Cancer

**DOI:** 10.1155/2011/819724

**Published:** 2011-12-22

**Authors:** Patrizia Pellegrini, Ida Contasta, Tiziana Del Beato, Fabiana Ciccone, Anna Maria Berghella

**Affiliations:** National Council of Research (CNR), Institute of Translational Pharmacology (IFT), 67100 L'Aquila, Italy

## Abstract

Studies focusing on gender have shown that differences exist in how the immune system responds to disease and therapy. Understanding how gender influences immunological mechanisms in health and disease and identifying gender-specific biomarkers could lead to specifically tailored treatment and ultimately improve therapeutic success rates. T helper1 (Th1) and Th2 cytokines (Th1/Th2) have pivotal roles in the homeostasis of Th1 and Th2 cell network functions in the immune response but sex steroids affect Th1/Th2 production in different ways and a natural sexual dimorphism in the immune response has been shown. In order to investigate these differences further, we developed Th-cytokine data-driven models of the immune response and evaluated healthy subject peripheral blood samples. Independent cohorts of colorectal cancer and adenoma patients were also studied for comparison purposes. Our results show that the interferon (IFN)**γ** production pathway for immune response homeostasis is specific to men whilst the interleukin- (IL-) 6 production pathway for immune response homeostasis is specific to women. The IL-10 pathway for restoring immune system resting homeostasis was common to both but was controlled by the respective gender-specific pathways. These gender pathways could well be used as targets and biomarkers in translational research into developing new clinical strategies.

## 1. Introduction

Advances in the understanding of pathological mechanisms and the identification of disease targets and biomarkers have had a considerable impact on clinical practice [1]. One change has been the shift from generalized medicine to a stratified approach, with patients being placed in clinical diagnostic or therapeutic subgroups according to specific biomarkers [2, 3]. It is hoped that this approach will lead to more specific and effective treatment in the not too distant future but this success depends upon the identification of specific biomarkers that can be measured easily from disease onset. Peripheral blood targets/biomarkers are currently the most practical, noninvasive means of diagnosing disease, predicting prognosis, and therapeutic response [4]. The identification of gender-specific biomarkers in peripheral blood would therefore open up an interesting field for research given gender-related susceptibility to disease [5]. Sex steroids, for example, have been shown to influence the regulation of Th cell network balance, shifting the balance toward a Th1 and/or Th2 type response, and both clinical and experimental data have demonstrated the presence of a natural sexual dimorphism in the immune response [5–8]. During their reproductive years, females have a more vigorous cellular and humoral immune response than males and a greater ability to reject tumors and homografts [9–14]. Evidence suggests that physiological levels of estrogen affect humoral and cell-mediated immune responses, while the male hormone, testosterone, does the opposite [15–17]. Ironically, this enhanced baseline immune function is associated with a higher prevalence of autoimmune disorders in females of reproductive age [6], than in postmenopausal women or men [18–21]. Sex steroids seem to affect Th1/Th2 production in different ways: during pregnancy, the Th1/Th2 network balance is skewed toward Th2 [22], thereby preventing rejection of the antigenically foreign fetus by a cell-mediated immune attack [23–26]. The *in vitro* influence of sex steroids on T-cell cytokine production has been studied extensively [27–30], showing, however, complex and diverse effects. 

 We believe that differences in Th1/Th2 production pathways in men and women are responsible for differences in the immune response in health and disease. Gender differences in immunological pathways imply different reactions to disease as well as different reactions to drugs and hence the identification of these gender-specific pathways could lead to more successful treatment.

In order to demonstrate these differences, we developed Th-cytokine data-driven models of the immune response and evaluated peripheral blood samples taken from healthy men and women. Independent cohorts of colorectal cancer and adenoma patients were also evaluated for comparison purposes. Our study indicates, for the first time, that gender-specific Th1/Th2 pathways operate in maintaining the homeostasis of the immunological cell network. These gender-specific pathways may well be responsible for differing gender-dependent responses to disease and therapy and open up an exciting new field for research.

## 2. Materials and Methods

### 2.1. Experimental Design

Human studies were performed in accordance with the standards of the Ethics Committee and all persons gave their informed consent prior to their inclusion in the study. To establish whether gender-specific Th1/Th2 cytokine production pathways could be at the basis of differences in immunological responses we designed an experimental approach based on the use of cytokine data-driven computational models of the immune response (Figure 4).

 Whole blood levels of Th1 and Th2 cytokines, indicative of Th1 or Th2 cell differentiation, were used (Figure 4); the relative proportion of each Th cell-type generation depends on the cytokines produced by APCs (cellular network) and released into the cell environment during resting and activation states of the immune response. Whole blood contains all blood cells and the cell environment and so it includes the cytokine levels from the “cellular network” and the “environment network”, reflecting *in vivo *physiological conditions more accurately and so appropriate to this study.

 We worked on the assumption that the network profile of the production levels of Th1 and Th2 cytokines (*level network profile*) reflected Th1 or Th2 differentiation: balance between the levels of Th1 and Th2 cytokine production indicated normal Th1 and Th2 cell differentiation and so a productive immune response. 

 We determined level network profiles in whole blood culture supernatants without activation (APC and T cells in resting conditions) and with LPS (activated APCs) and PHA (activated T cells). We also analyzed (i) the PHA-level network profiles of separated (Ficoll/Hypaque gradient) peripheral blood mononuclear cells (PBMCs), in order to discover whether T cellular components affect Th1/Th2 interaction; and (ii) the level network profiles in blood serum, to identify gender-specific Th1/Th2 biomarkers. The level network profiles were also determined in colorectal cancer and colon adenoma patient groups divided by sex, as independent cohorts for comparison purposes. 

 The cytokines used in our Th-cytokine data-driven computational models of the immune response were as follows: IL-2, IFN*γ*, IL-4, IL-6, and IL-10, to make up our basic network model, to establish if the direction in T cell differentiation was Th1 and/or Th2 type; tumor necrosis factor (TNF) *α* and IL-1*β* as serum biomarkers and IFN*γ*, IL-6, and IL-10 as LPS whole blood biomarkers, to determine if antigen presenting cells (APCs) direction on T cell differentiation was of Th1 or Th2 type; and soluble (s) IL-2 receptor (R) and sIL-6R to estimate cell activation. Indeed, IL-2 and IFNy support Th1 functions [31] promoting cell-mediated immunity; IL-4, IL-6, and IL-10 are associated with Th2 responses and IL-10 is a powerful inhibitor of IFN*γ* and macrophages [32]. IL-6 also supports Th17 functions, suppressing Th1 function [33, 34], and has a key function in homeostasis influencing Th differentiation into T regulatory (Treg) or Th17 cell subsets. TNF*α* and IL-1*β*, on the other hand, are some of the key mediators produced by APCs that dictate the course of immune responses. sIL-2R and sIL-6R are activation markers [35, 36]. 

### 2.2. Healthy Subjects

A group of 66 healthy subjects were studied (33 men and 33 women). None of the subjects were receiving concurrent drug treatment including widely used pharmaceuticals, such as salicylates and sex hormones (contraceptive pill, hormone replacement therapy). Distribution of age in the male and female groups was the same (men: *N* = 33 mean ± SD = 41 ± 12.00 years; women: *N* = 33 mean ± SD = 41 ± 15.00 years; *P* = 0.14).

### 2.3. Independent Validation Cohorts of Colorectal Cancer and Adenoma Patients

A group of 110 patients, 64 men and 46 women, who were diagnosed for the first time as having colorectal cancer and had to undergo colectomy were studied. Distribution of age in male and female groups was the same (men: mean ± SD = 65.60 ± 10.90 years; women: mean ± SD = 65.90 ± 10.40 years; *P* = 0.89). Clinical diagnosis was confirmed histopathologically and patients were subtyped using the pTNM classification (according to the diagnostic criteria of the American Joint Committee on Cancer and the Committee of the International Union Against Cancer), as follows: men 16 stage I, 30 stage II, 8 stage III, 10 stage IV; and women 4 stage I, 23 stage II, 13 stage III, and 6 stage IV. None of the patients received radiation or chemotherapy before surgery. Distribution of stage in male and female groups was the same (*P* = 0.87). Tumors varied from 2.5 to 9.0 cm in diameter.

 A group of 8 colon adenoma patients, 4 men and 4 women, were also studied. Distribution of age in the male and female groups was the same (men: mean ± SD = 64.75 ± 4.99 years; women mean ± SD = 69.50 ± 13.02 years, *P* = 0.52). Clinical diagnosis was confirmed histopathologically. Distribution of age in male and female groups between colorectal cancer and adenoma patients was also the same (*P* = 0.61).

### 2.4. Blood Samples

Blood was collected at the same time of day to minimize the effects of diurnal variation. A 15 mL sample of heparinized (Liquemin-Roche) blood (20 IU heparin/mL blood) was taken from each subject, and the samples, kept at room temperature, were used immediately in whole blood cell cultures. Additionally, 5 mL sample of peripheral blood without heparin was also taken and, within 1 h of withdrawal, the serum was stored in aliquots at −80°C until use.

### 2.5. Whole Blood Cell Cultures

Heparinized venous blood [37] was diluted 1 : 10 with RPMI-1640 medium (Sigma, endotoxin tested), which was supplemented with L-glutamine 0.2 mM, penicillin 50 IU/mL, and streptomycin 50 *μ*g/mL (Sigma) and distributed in 0.5 mL aliquots in 12 mm polystyrol tubes. 10 *μ*g/mL of PHA and 10 *μ*g/mL of LPS (Sigma) were used for stimulation; aliquots without stimuli were also prepared. Cell cultures were incubated at 37°C in a humidified atmosphere of 5% CO_2_. After 24 h and 72 h of culture without a change of medium, 320 *μ*L supernatant was removed from each tube to be assayed for cytokine levels and stored in aliquots at −80°C until used. The effect of adding heparin which prevents clotting in whole blood cultures was tested (data not shown). 

 Experimental conditions were as follows: with stimuli (+PHA and +LPS) in order to recreate an activation situation and without stimuli to evaluate immune response in resting conditions. PHA was used to study T cell contribution [38] and LPS the influence of antigen presenting cells [39]. As mentioned in the “experimental design” the whole blood culture method was used [40]. It is a simple procedure and thus reduces the potential for error and offers the added advantage of not requiring the separation of cellular subpopulations which would represent an additional source of potential variation both in individuals and among individuals.

### 2.6. PBMC Cell Cultures

PBMCs were separated by centrifugation over a Ficoll/Hypaque gradient (20 min, 1000 ×g) and washed with RPMI-1640 medium (Gibco). Isolated cells were cultured at a concentration of 1 × 10^6^ cells/mL in RPMI-1640 complete medium (supplemented with 10% fetal calf serum, L-glutamine 0.2 nM, penicillin 50 UI/mL, streptomycin 50 *μ*g/mL; Sigma). Supernatants were obtained from PBMC cultures in RPMI-1640 complete medium. The cells (with and without PHA, 3 *μ*g/mL) were incubated at concentrations of 1 × 10^6^ cells/mL at 37°C in a humidified atmosphere of 5% CO_2_. After 24 h of culture without a change of medium, 120 *μ*L supernatant was removed from each well, centrifuged at 250x g, and stored in aliquots at −80°C until use.

### 2.7. Cytokine Detection

ELISA assays were used. This method has been described in detail elsewhere [41]. For intraassay precision, standard samples of known cytokine concentrations were assayed in replicates of 10, and the coefficient of variation was <10%. For interassay precision standard samples were assayed 30 times in multiple assays to determine precision between assays, and the coefficient of variation was <10%.

The sensitivity of these ELISA assays was as follows: sIL-2R <50 IU/mL, TNF*α* <1.5 pg/mL, (T Cell Diagnostics-Cambridge, USA), IL-1*β*, IL-2 <5 pg/mL, and IL-4 <1 pg/mL (Endogen, Cambridge, USA); sIL-6R <4, 3 ng/mL (Biosource, Belgium); IL-6 <2 pg/mL, IFN*γ* <4 pg/mL, and IL-10 <5 pg/mL, (Benfer-Scheller, Keystone, USA). Cytokine values were obtained using a specific software program (ELISA-AID, Eurogenetics).

### 2.8. Statistical Analyses

In physiological systems components operate as a network and individual network components vary dynamically and covary with respect to one another. Therefore, the identification of Th-cytokine physiological pathways in this study and correlated biomarkers can only be achieved through evaluations that take into account systems biology characteristics [42, 43]. This entails determining the level of cytokines, the study of the relationships between cytokine levels, and then the behaviour of this multicomponent system as a network. Due to the complexity of biological systems, this requires the use of mathematical models that provide a framework for determining the outcome of numerous and simultaneous time-dependent and space-dependent processes [44–46]. Hence, in addition to the study of statistical differences between Th1 and Th2 cytokines, using the Mann-Whitney *U* test or the Student's *t*-test (as appropriate), we studied data-driven Th1/Th2 cytokine models through multivariate statistical analyses using “Statgraphics software systems” (full system 5.25 version 4.0; graphics system by statistical graphics corporation ed., USA, 1989). Values of *P* ≤ 0.05 were considered significant.

 We used the multivariate statistical procedure that analyses the correlation between parameters and produces a matrix of correlation coefficients (that vary from −1 to +1) and significance (*P*), allowing a dynamic analysis of how network components vary with respect to one another at any moment in time. A positive correlation indicates that the parameters vary in the same direction, while negative correlation indicates that the parameters vary in the opposite direction. In fact, the multivariate statistical procedure that analyses the correlation measures the linear associations between all parameters, and if parameters increase or decrease at the same time, the correlation is positive, whilst other changes are considered negative. Statistically independent parameters have an expected correlation of zero. 

 The multiple regression analysis, which provides a modeling technique that allows us to relate a dependent variable to one or more independent variables, was also used. Regression analysis allows us to summarize data and quantify the nature and strength of the relationships among variables. Hence, the multiple regression and stepwise multiple regression analyses (which assume that a variable can be predicted from a set of other variables and seek the best mathematical relationship between them) were used to study the weight of each cytokine in the normal balance of Th1/Th2 physiological network. This procedure may be helpful in building a model when we have a large number of possible independent variables and are unsure which to include. A forward or backward selection procedure is possible in the latter method. The forward selection begins with no variables (step 0) and adds them one at a time (steps 1, 2, etc.) according to the highest *F*-statistic values. This allows us to control the entry of variables into the model. The backward selection procedure begins with a model containing all the variables (step 0) and eliminates them one at a time (steps 1, 2, etc.) according to the lowest *F*-statistic values. The forward selection is comparable to onset and evolution of the immune response whilst the backward selection procedure is comparable to the physiological return to equilibrium. When we have finished entering and removing variables, the system then estimates the final model using the Graham-Schmidt algorithm to get the most accurate estimates possible and display the model fitting results.

## 3. Results

### 3.1. Healthy Subjects: Differences in the Level Network Profiles of Men and Women Are Not, in Theory, Responsible for the Sexually Dimorphic Generation of the Immune Response in Healthy Subjects

Pathological conditions have been found to arise from alterations in the environment Th1/Th2 cytokine network since the relative proportion of each Th1 and/or Th2 cell-type generation, and so the type of immune response, depends on the level of each Th1 and/or Th2-type of cytokine. Our results show that gender-related differences in the immune response in health are not the result of differences between male and female level network profiles, because no significant differences were observed in these profiles, with the exception of IL-10 which was higher in men when PHA stimulus was used (see Table 1).

### 3.2. Differences in the Relationships in the “Level Network Profiles” However, Could Be Responsible for the Sexual Dimorphism of the Immune Response in Health

**Figure 1:**
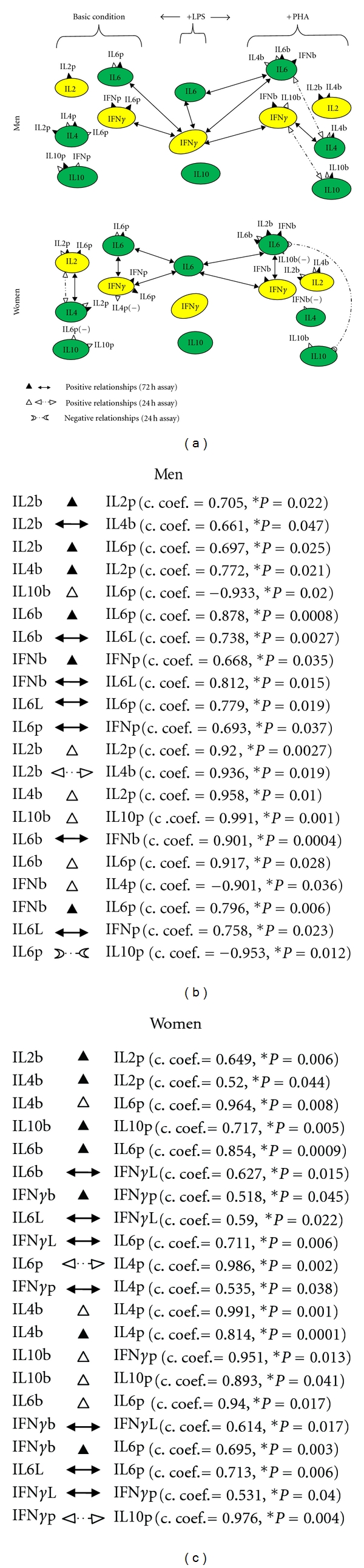
Relationships using the whole blood assay method (72h assay, black): (black triangle) Positive relationships. Relationships using PBMC cells separated by Ficoll/Hypaque gradient procedures (24 h assay, white): (white triangle) positive and (crescent shape) negative relationships. Basic Condition: resting state; +LPS: APC activated condition; +PHA: T cell activated condition. Basic condition (b), LPS (L), PHA (p). In healthy subjects, differences in the relationships in the “level network profiles” could be responsible for the sexual dimorphism of the immune response in health. Gender-specific relationships in “level network profiles” affect the direction (Th1 or Th2) of the immune response under resting (Basic) and activated (+PHA) conditions. APCs (**+LPS**) regulate the starting type and evolution of immunological responses in both men and women: the starting type (+LPS → Basic,) appears to be regulated by IFN*γ* production in men, and by IL6 in women; the evolution (+LPS → +PHA) by continuing IL6 production in women and by IFN*γ* in men. No significant relationships were found in whole blood culture supernatants without stimulus (basic conditions) in men. In women, this regulation would appear to be exerted by the linked production of IL2, IL4 and IFN*γ*, IL6 cytokines (relationships in basic conditions). The IL2 and IL4 interregulation in women seems to have both an early and late role since the correlation between their levels are significant in both the 24h and 72h culture supernatant cytokine assays; the interregulation between IL6 and IFN*γ* levels only has a late function because it is only significant in the 72h assay. The earlier evolution of activated T cells (+PHA 24h culture) seems to be influenced by the linked production of IL6 and IL4, IFN*γ* and IL10 cytokines in men; while by IL6 and IL10 cytokines in women. The late evolution of activated T cells (+PHA 72h whole blood of culture) on the other hand seems to be influenced by the linked production of IFN*γ* and IL4 in men, while by IFN*γ* and IL6 in women.

In fact, in the evaluation of the Th-cytokine data-driven models of the immune response (Figure 4) it emerged that the level network profile with activated APCs (+LPS, Figure 1) affects the direction of the immune response in both men and women under resting (Basic, Figure 1) and activated (+PHA, Figure 1) conditions. 

 Hence, APCs (+LPS, Figure 1) regulate the starting type (Basic) and evolution (+PHA) of immunological responses in both men and women, but the effect under resting conditions (Basic, Figure 1), resembling the onset of the immune response (because the cells are in the resting state), appears to be exerted by IFN*γ* production in men, and by IL-6 in women, whilst in activated conditions (PHA, Figure 1), resembling the evolution of the immune responses (because the cells are in the activation state), by IL-6 production again in women but by IFN*γ* and IL-6 in men. 

 The network profile of the production levels of Th1 and Th2 cytokines (*level network profile*) in resting (Basic, in the sense of unstimulated) conditions does not appear to have a specific role in T cell differentiation in men since no significant relationships were found in whole blood culture supernatants without stimulus (Figure 1). In women, this regulation would appear to be exerted through a Th1 and Th2 linked production of IL-2 (Th1) and IL-4 (Th2), IFN*γ* (Th1) and IL-6 (Th2) cytokines (Figure 1). Interestingly, this IL-2 and IL-4 Th1/Th2 interregulation in women seems to have both an early and late role in the control of the Th1 and Th2 cell network since the relationships between their levels are significant in both the 24-hour and 72-hour culture supernatant cytokine assays. The interregulation between IL-6 and IFN*γ* levels is only significant in the 72-hour assay (cytokine assay of the whole blood supernatant after 72 h of cell culture). 

 Additionally, in Figure 1 it would appear that the early differentiation of activated T cells (+PHA after 24 h of culture) is influenced by the positive linked production of IL-6 and IL-4, IFN*γ* and IL10 cytokines in men, and the negative linked production of IL-6 and IL-10 cytokines in women. Likewise the late Th1 or Th2 differentiation of activated T cells (+PHA after 72 h of culture) seems to be influenced by the positive linked production of IFN*γ* and IL-4 in men, while by IL-6 and IFN*γ* in women.

### 3.3. Differences between Men and Women in the Relationships of Serum “Level Network Profiles” Could Represent Possible Gender Biomarkers for Sexually Dimorphic Generation of Immune Responses in Health and Disease States

**Figure 2:**
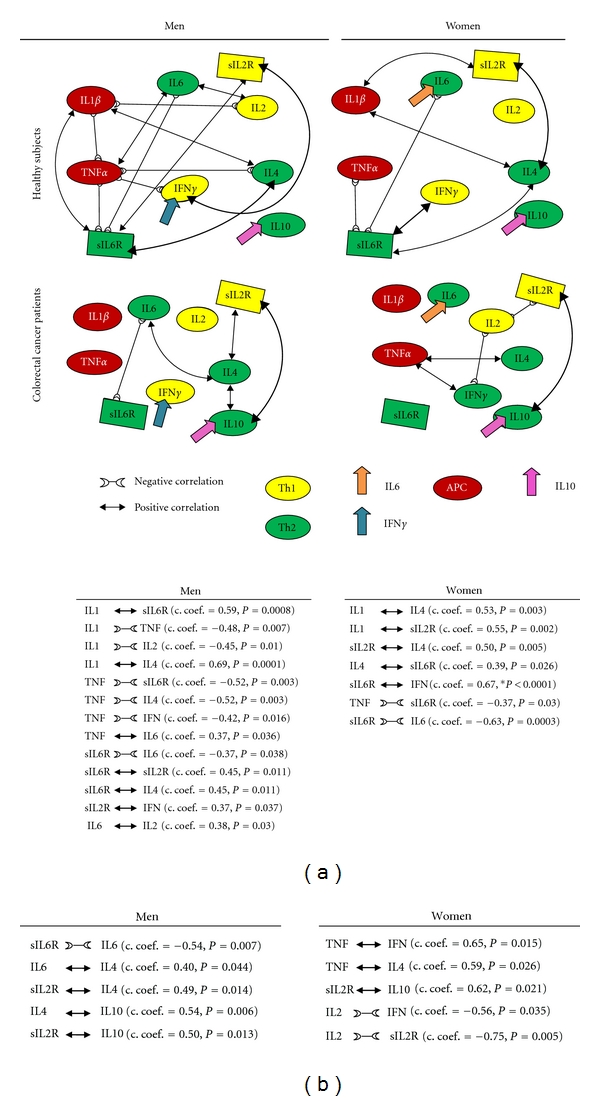
Differences between men and women in the relationships of serum “level network profiles” could represent possible gender biomarkers for sexually dimorphic generation of immune responses in health and disease states. In healthy subjects (a), significant gender-specific and gender-common Th1/Th2 network relationships were found in serum which could be used as biomarkers to identify the direction of T cell differentiation. However, in neither sex did the IL10 cytokine interact with other network components. In colorectal cancer patients (b) no significant relationships with IFN*γ* in the male group were observed or with IL6 in the female group, indicating alterations in the gender-specific Th-cytokine pathways; significant relationships between IL10 and other Th1/Th2 network components were observed in both men and women groups indicating alterations in the gender-common pathways but through different Th1/Th2 pathways.

Gender-specific and gender-common significant Th1/Th2 network relationships were found in serum in men and women (Figure 2). A gender-specific biomarker in resting conditions (Figure 4) may be the positive relationship between IL-2 and IL-6 cytokines which was significant in men but not women (Figures 2 and 5: “T cell”). Gender-specific biomarkers in activation conditions (Figure 4) may be the positive relationships between sIL-2R and IFN*γ*, sIL6-R, and sIL-2R (Figures 2 and 5: “T cell”) which again were significant in men, but not in women. No women gender-specific relationships were found in resting conditions but positive relationships between sIL-2R and IL-4, sIL-6R and IFN*γ* were identified in activation conditions (Figure 5) that could be used as biomarkers. The positive relationship between sIL-6R and IL-4 and the negative one between sIL6R and IL6 may represent common activation biomarkers for both men and women.

 The negative relationships between IL-1*β* and IL-2, TNF*α* and IL-1*β*, TNF*α* and IFN*γ*, and TNF*α* and IL-4 and the positive one between TNF*α* and IL-6 (Figures 2 and 5: “APC”), are possible male gender specific biomarkers for APC T cell differentiation in men in resting conditions (Figure 5). There were no female APC gender-specific biomarkers in resting conditions, whilst a common biomarker in resting conditions appears to be the positive relationship between IL-1*β* and IL-4 (Figures 2 and 5). Finally, the positive relationships between IL-1*β* and sIL6R in men and IL-1*β* and sIL-2R in women (Figure 2) are possible gender-specific biomarkers for APC T cell differentiation in activated conditions, whereas the negative relationship between TNF*α* and sIL-6R a common APC biomarker (Figure 5).

 Additionally age in men was related to IL-1*β* (c.coef. = 0.45, *P* = 0.010) and in women to IFN*γ* (c.coef. = 0.71, *P < 0.0001), sIL-6R (c.coef. = 0.51, *P* = 0.004) and IL-10 (c.coef. = −0.35, *P* = 0.047).

### 3.4. Colorectal Cancer and Adenoma Patients: Differences between Men and Women in “Level Network Profiles” Are Not Responsible for the Sexually Dimorphic Generation of Immune Responses in Disease but as Discussed in the Last Section, Differing Responses May Arise from Differences in the Relationships within “Level Network Profiles”

**Figure 3:**
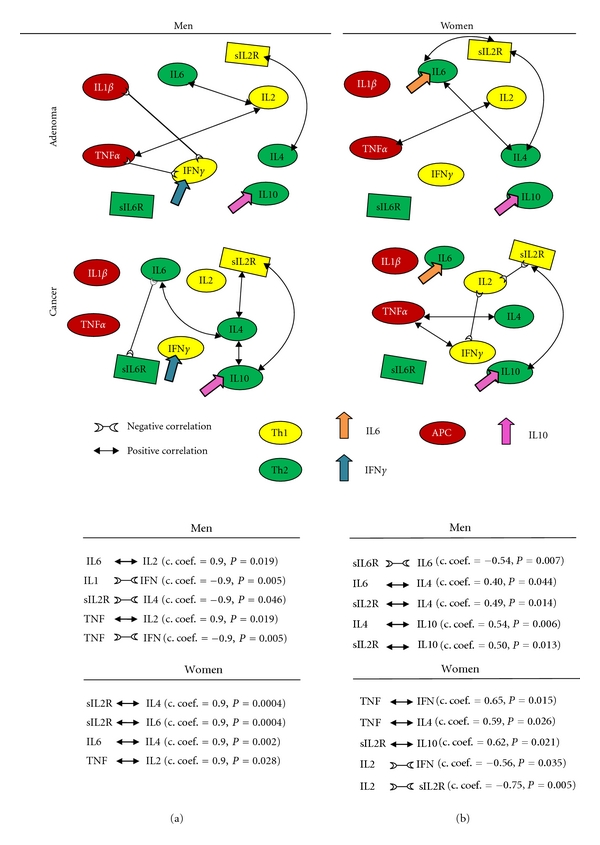
In adenoma patients (a), gender-specific pathways partially regulate Th1 and Th2 cell network homeostasis. In neither sex was a significant relationship observed between IL10 and other Th1/Th2 network components. IFN*γ* and IL6 pathways (in men and women, resp.) still regulate, albeit partially, the sex-specific Th1 and Th2 cell network homeostasis (and so the immune response) in adenoma patients; in neither sex was a significant relationship observed between IL10 and other Th1 and Th2 network cytokines. No significant relationships for IFN*γ* or IL6 (in men and women, resp.) were observed in colorectal cancer patients (b), indicating alterations in the gender-specific regulatory pathways responsible for Th1/Th2 physiological homeostasis. The persistence of IL10 within the environmental network is a significant biomarker for the loss of Th1 and Th2 cell network homeostasis and disease progression in both men and women, mediated however through different sex-related Th1/Th2 pathways. In normal immune response the influence of IL10 on Th polarization is short-lived in both sexes.

In order to confirm our results on healthy subjects, independent cohorts of colorectal cancer and adenoma patients were also assessed using the same Th-cytokine data-driven computational models. No significant differences were found between men and women in serum “level network profiles” in both colorectal cancer and adenoma patients, confirming our results concerning healthy subjects (Table 1). In addition, significant alterations in the IFN*γ* and IL-6 gender-specific pathways and IL-10 gender-common pathways were found in colorectal cancer patients (Figure 2). Further confirmation also came from the finding that in adenoma patients, gender-specific pathways IFN*γ* and IL-6 still partially regulate immune response homeostasis in men and women and in neither sex was a significant relationship observed between IL-10 and the other Th1/Th2 network components (Figure 3).

### 3.5. Colorectal Cancer Patients: Alterations in the Relationships of IFN*γ* and IL-6 Gender-Specific Pathways and of IL-10 Gender-Common Pathways Are Biomarkers for the Loss of Immune Response Homeostasis and Disease Progression in Both Men and Women, but through Gender-Specific Mechanisms

**Figure 4:**
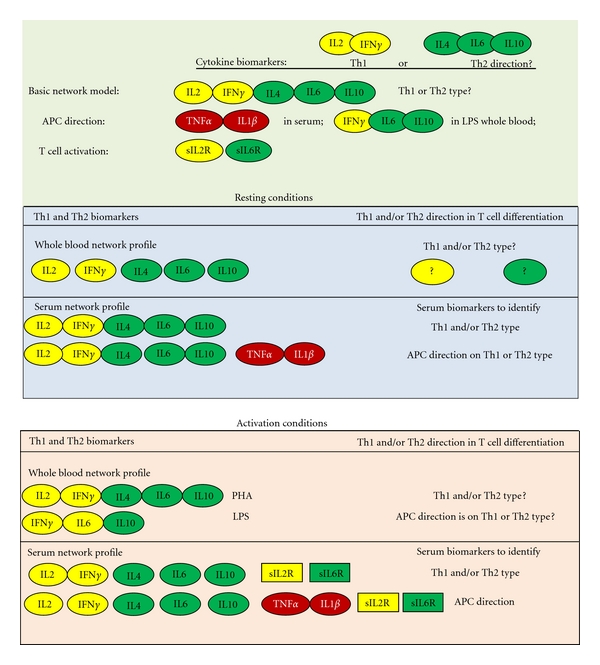
Th-cytokine models of the immune response in resting and activation conditions: whole blood levels of specific Th1 and Th2 cytokines were used as biomarkers in Th-cytokine data-driven computational models of the immune response to determine the direction of T cell differentiation (Th1 or Th2). The cytokines used in our Th-cytokine data-driven computational models of the immune response were: interleukin (IL)-2, interferon (IFN)*γ*, IL-4, IL-6, and IL-10, to make up our basic network model, to establish if the direction in T cell differentiation was Th1 and/or Th2 type; tumor necrosis factor (TNF)*α* and IL-1*β* as serum biomarkers and IFN*γ*, IL-6, and IL-10 as LPS whole blood biomarkers, to determine if APC direction on T cell differentiation was of Th1 or Th2 type; and soluble (s) IL-2 receptor (R) and sIL-6R to estimate cell activation. APC: antigen presenting cells; PHA: to study T cell network contribution; LPS: to evaluate the influence of antigen presenting cells.

**Figure 5:**
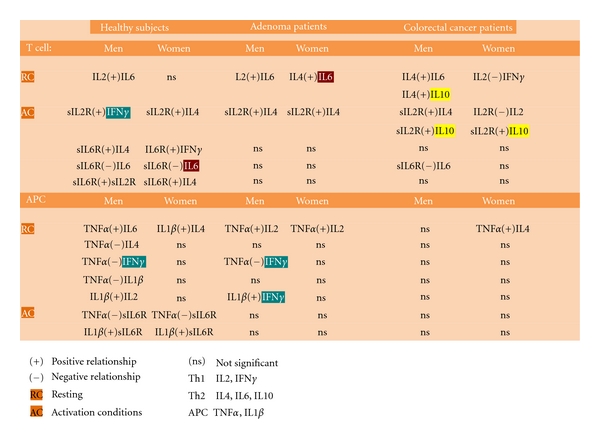
Serum Th1 and Th2 network relationships. Significant gender common and gender-specific Th1/Th2 network relationships were found in serum which could be used as biomarkers to indicate the direction of T cell differentiation in the immune response. Positive relationship (+); negative relationship (−); resting (RC) and activation (AC) conditions; not significant (ns); Th1: IL2, IFN*γ*; Th2: IL4, IL6, IL10; APC: TNF*α*, IL1*β.*

No significant relationships with IFN*γ* were observed in the male group or with IL-6 in the female group indicating alterations in the gender-specific Th-cytokine pathways (Figure 2, healthy subjects and colorectal cancer patients). Significant relationships between IL-10 and other Th1/Th2 network components were observed in both men and women groups, but through different Th1/Th2 pathways, indicating alterations in gender common Th-cytokine pathways. However, sexual dimorphism in cytokine relationships included (Figures 2 and 5) the following: (1) a positive relationship between serum levels of IL-6 and IL-4 in the male group, which in its turn was positively correlated to IL-10 levels; (2) a negative relationship between IL-2 and IFN*γ* in the female group; (3) positive relationships between serum levels of sIL-2R activation biomarker and IL-10 in men, in addition to the relationship between sIL-2R and IL-4; and (4) a negative relationship between sIL-2R and IL-2, and a positive one between sIL-2R and IL-10 in the female group. Finally, positive relationships between TNF*α*, the APC biomarker, and IFN*γ* and IL-4 were again found in the female group (Figure 2). However, no relationships between APC biomarkers in activation conditions (Figure 4) were found in either group (Figures 2 and 5). 

 In fact, we found that patient disease progression (stage correlation) was related to an increase of IL-10 (men: c.coef. = 0.61, *P* = 0.002; women: c.coef. = 0.81, *P* = 0.002) and sIL-2R (men: c.coef.=0.39, *P* = 0.048; women: c.coef.=0.70, *P* = 0.009) ) in both sexes, but in men disease progression is also related to an increase of IL-4 (c.coef. = 0.49, *P* = 0.014) and IL-6 (c.coef. = 0.42, *P* = 0.034), while in women to a decrease in IL-2 (c.coef. = −0.58, *P* = 0.031). Moreover in women age is linked to a decrease of TNF*α* (c.coef. = −0.67, *P* = 0.012) and sIL-2R (c.coef.= −0.57, *P* = 0.033).

### 3.6. Adenoma Patients: The Relationships Described Represent Gender-Specific Biomarkers for the Passage from Health to Adenoma and Colorectal Cancer Disease

Gender-specific IFN*γ* (men) and IL-6 (women) pathways still partially regulate Th1 and Th2 cell network homeostasis in adenoma patients, in contrast to colorectal patients (Figure 3). Under immune resting conditions (Figure 4), the significant positive relationship between IL-6 and IL-4 indicates that IL-6 pathways were still operating within the Th1/Th2 network in the group of female patients (Figures 3 and 5). No significant Th1 polarization biomarkers were found in either sex under activation conditions; Th2 polarization biomarkers, on the other hand, were linked to an increase of sIL-2R and IL-4 in men, and sIL-2R and IL-6 plus sIL-2R and IL-4 in women (Figures 3 and 5).

 Under immune resting conditions (Figure 4) the significant negative relationship between TNF*α* and IFN*γ*, and the positive one between IL-1*β* and IFN*γ* levels, in addition to TNF*α* and IL-2, indicate that APC IFN*γ* pathways were still operating within the Th1/Th2 network model under basic conditions in men (Figures 3 and 5). In women, the influence of APCs under basic conditions emerges from positive relationship between TNF*α* and IL-2 (Figures 3 and 5). APCs do not seem to influence Th1 and Th2 cell network homeostasis under activation conditions (Figure 4). In fact, no statistically significant relationships were found between soluble molecules (sIL-2R and sIL-6R) and TNF*α* or IL-1*β* in either group (Figures 3 and 5).

 Even if the results of the adenoma study should be handled with prudence considering the number of patients, IFN*γ* and IL-6 pathways partially regulate Th1 and Th2 cell network homeostasis (IFN*γ* in men and IL-6 in women, resp.). However, in neither sex was a significant relationship observed between IL-10 and other Th1/Th2 network component which should be short-lived in both sexes. Therefore, IL-10 environment persistence is a biomarker for the loss of the regulatory mechanisms responsible for restoring the initial Th1/Th2 physiological equilibrium [47] in men and women. 

## 4. Significant Independent Factors for Predicting Alterations in Immune Response Homeostasis Regulation of Common and Gender-Specific Th-Pathways

**Figure 6:**
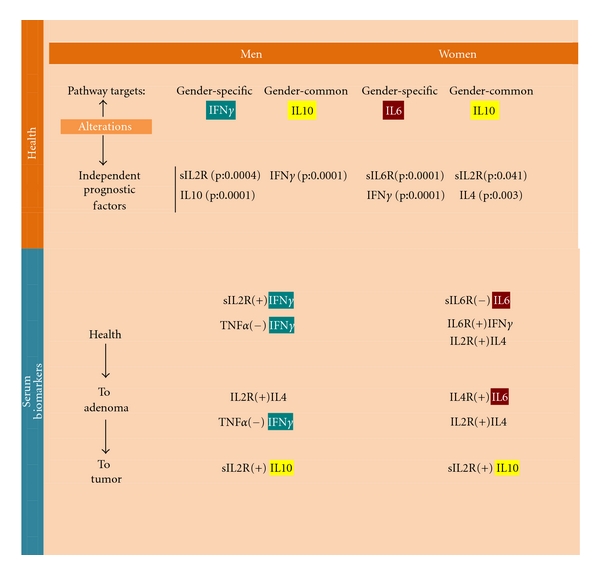
The results of the stepwise multiple regression analysis (using the forward procedure) indicate that the serum level of IFN*γ* in men could be a significant independent factor for predicting a possible alteration in IL10 regulation of the balance between Th1 and Th2 cell types. The independent factors sIL2R and IL10 are important for predicting an alteration in the normal regulation that IFN*γ* exerts over the balance between Th1 and Th2 cell types. In women sIL2R and IL4 may prove useful as significant independent factors to predict alterations in the normal regulation that IL10 exerts over the balance between Th1 and Th2 cell types; likewise sIL6R and IFN*γ* may prove useful as significant independent factors to predict alterations in the normal regulation that IL6 exerts over the balance between Th1 and Th2 cell types.

The stepwise multiple regression analysis, using the forward procedure, allowed us to identify the greatest weighting parameters on IFN*γ* and IL-6 gender-specific pathways and IL-10 gender-common pathways. The results also indicate that the serum level of IFN*γ*  (*P* = 0.0001) in men could be a significant independent factor for predicting a possible alteration in IL-10 regulation of the balance between Th1 and Th2 cell types (Figure 6). The independent factors sIL-2R (*P* = 0.0004) and IL-10 (*P* = 0.0001) are, on the other hand, important for predicting an alteration in the normal regulation that IFN*γ* exerts over the balance between Th1 and Th2 cell types. In women (Figure 6) sIL-2R (*P* = 0.041) and IL-4 (*P* = 0.003) may prove useful as significant independent factors to predict alterations in the normal regulation that IL-10 exerts over the balance between Th1 and Th2 cell types; likewise sIL-6R (*P* < 0.0001) and IFN*γ* (*P* < 0.0001) may prove useful as significant independent factors to predict alterations in the normal regulation that IL-6 exerts over the balance between Th1 and Th2 cell types. The results of multiple regression analysis show that age could also be a significant independent factor for IFN*γ* (*P* = 0.01) and IL-10 (*P* = 0.03) in men; whilst in women age appears to be significant for sIL-6R (*P* = 0.002) and IFN*γ* (*P* = 0.04).

## 5. Discussion

We put forward the hypothesis that gender-dependent immune responses in health and disease states and differing reactions to disease and therapy could be due to gender-specific Th1/Th2 production pathways. The identification of these gender-specific pathways and the correlated targets/biomarkers could lead to more specifically tailored treatment and better therapeutic success rates. In order to test this hypothesis, we decided to study and evaluate the possibility of using Th1 and Th2 cytokines as biomarkers in immune response models, as they are responsible for propelling the immune response in a given Th1 or Th2 direction: the “level network profile” (the network profile of the production levels of Th1 and Th2 cytokines) by APCs is indicative of the direction of T cell differentiation during the immune response, and the balance between their levels and between their relationships indicates a normal Th1 and Th2 cell differentiation and so a productive immune response; a lack of balance indicates pathology. We developed Th-cytokine data-driven models of the immune response (Figure 4) and evaluated peripheral blood samples of healthy subjects. To back up our results, independent cohorts of colorectal cancer and adenoma patients were also evaluated. Our hypothesis was confirmed since our results not only indicate that gender-specific treatment should improve therapeutic success rates but also highlight the importance of peripheral blood Th1/Th2 network pathways as physiological targets/biomarkers in clinical investigations and translational pharmacology research. 

 The results of this study indicate, for the first time, that physiological gender-specific Th1/Th2 pathways regulate the homeostasis of the Th1/Th2 cell network and hence the immune response. These gender pathways are therefore probably responsible for gender-dependent reactions to disease and therapy as a consequence of their specific, regulatory roles in Th cell polarization during the development of the immune response and in restoring physiological homeostasis.

 The new points that emerge from our study can be summed up as follows: (1) IFN*γ* and IL-6 production pathways are respectively male and female gender-specific health pathways for immune response homeostasis (Figure 1) and consequently targets and/or biomarkers for the passage from health to adenoma and colorectal cancer (Figures 2 and 3); (2) the IL-10 pathway is a common-gender pathway involved in restoring immune system resting homeostasis (Figure 1), but only if controlled by the respectively gender specific pathways; otherwise it is a cancer progression target/biomarker (Figures 2 and 3); (3) the gender specific differences in serum “level network profiles” represent significant biomarkers that could be used to develop more specific approaches (Figures 5 and 6). 

 In more detail, our results showed that gender specific IFN*γ* and IL-6 pathways respectively regulate male and female immune response homeostasis, however in neither sex were significant relationships observed between IL-10 and other Th1/Th2 network components, apart from that between IL-10 and IFN*γ* in the male group and IL-10 and IL-6 in the female group precociously in the cellular network (24 h) (Figure 1). In order to maintain a normal balance between Th1 and Th2 cells, the effect of IL-10 on Th polarization must be, therefore, short-lived and in the linked positive production of IFN*γ* and IL-10 cytokines in men and the negative production of IL-6 and IL-10 in women, IFN*γ* and IL-6 pathways could be considered gender specific pathways for the regulation of immune system homeostasis (Figure 1). APC regulation in Th1 and Th2 cell network homeostasis also appeared to be exerted through IFN*γ* production in men, and IL-6 production in women in both resting and activated conditions (Figure 1, correlation between the results of LPS and basic and PHA stimulus, resp.). So it would appear that IFN and IL-6 Th-cytokine pathways are gender specific targets and biomarkers for the onset and development of the immune responses, whilst IL-10 Th-cytokine pathways operate in the same way in both sexes, regulating the recovery of homeostatic equilibrium within the Th1 and Th2 cell network at the end of the immune response. 

 The above results in healthy subjects were confirmed by the results in the adenoma and colorectal cancer disease groups. In fact, within our colorectal cancer patient group we noted alterations in the IFN*γ* pathways in men and IL-6 in women and persistence of IL-10 under both resting and activated conditions (Figure 2). In the adenoma group, on the other hand, IFN*γ* and IL-6 pathways still partially regulated gender specific Th1 and Th2 cell network homeostasis but in neither sex was a significant relationship observed between IL-10 and other Th1/Th2 network components (Figure 3). 

Our results indicate that in the normal mucosa through adenoma to tumor progression, the host immune response proceeds from a physiological condition, where gender-specific Th1/Th2 pathways regulate the homeostasis of the Th1/Th2 cell network, to a type with partial or absent gender-specific Th1/Th2 pathways regulation and immunological suppressive characteristics (adenoma and cancer patients). Moreover, in the adenoma patients there was no IL-10 involvement, while this parameter was implicated in the cancer patients' immune responses, suggesting that IL-10 may be prognostic for the passage from adenoma to cancer as a dual biomarker together with sIL-2R (Figures 1, 2, 3, and 6). 

 In fact in healthy subjects, the sIL-2R in men and sIL-6R in women were principally related to IFN*γ* (Figures 2 and 6), which plays an important role in the development of Th1 cells; in adenoma the TNF*α* in men and IL-4 in women were, respectively, related to IFN*γ* and IL-6 (Figures 3 and 6). So in the adenoma patients, gender-specific Th1/Th2 pathways were involved in the Th1/Th2 network, while IL-10 (immunologically suppressive) was excluded; in the patient group there was an inverted situation.

Since the stepwise nature of colorectal cancer has been well defined and colon adenoma has been identified as a precursor of colorectal cancer, colon adenoma is a particularly meaningful intermediate outcome for studying factors related to colorectal cancer. Therefore, the differences observed between colon adenoma and colorectal cancer patients confirm that the IFNy production pathway for immune response homeostasis is specific to men, while the IL-6 production pathway for immune response homeostasis is specific to women. The IL-10, pathway for restoring immune system resting homeostasis was common to both but was controlled by the respective gender-specific pathways. In this way our hypothesis is confirmed: gender-dependent immune responses in health and disease states and differing reactions to disease and therapy could be due to gender-specific Th1/Th2 production pathways. These gender-specific pathways and the correlated targets/biomarkers (Figure 6) could lead to more specifically tailored treatment and better therapeutic success rates.

In fact, the observations made in this study may be useful for gender-specific therapeutic strategies. In men (Figures 2 and 6) changes in the level of IFNy, IL-10, and sIL-2R within the physiological normal ranges and high levels of the dual target sIL-2R/IFNy are biomarkers of immunological homeostasis and therapeutic success. Instead, significantly high levels of the dual target sIL-2R/IL-10 are biomarker of immune deficiency and treatment failure. Likewise in women (Figures 2 and 6) changes in the levels of sIL-6R, IFNy, sIL-2R, IL-4, and IL-10 within the physiological normal ranges and high levels of the dual targets sIL-6R/IFNy and sIL-2R/IL-4 are biomarkers of immunological homeostasis and therapeutic success. Instead, significantly high levels of the dual target sIL-2R and IL-10 are biomarker of immune deficiency and treatment failure.

The mechanisms responsible for gender-specific disease susceptibility have yet to be clarified. However our data suggest that the answer may lie in the differing capacity of cells to defend themselves against oxidative stress [48]. The cells of men and women differ greatly in terms of reactive oxygen species production and oxidative stress susceptibility [48–50] and this appears to be a promising new field of investigation. In all cell types it has been found, for example, that oxygen metabolism can lead to the production of reactive oxygen species (ROS) such as radicals. All cell types, including lymphocytes and other immune system cells, present a complex range of antioxidant compounds and enzymes, such as glutathione (GSH) and thioredoxin reductasi (TRX) [51, 52] to neutralize ROS and to preserve the cell oxidative balance. Gender-associated redox features of cells have also been described [49, 50]. The activities of ROS, for example, appear to be regulated differently in males and females and can be directly influenced by sex hormones [49, 50].

*In vivo* studies have further demonstrated the incapacity in males, but not in females, of maintaining intracellular reduced redox conditions, essential for normal cellular functions [48]; this explains, at least in part, the differences between the two sexes in the maintenance of the immune system homeostasis which we observed. In fact, if as has been proposed, IFN*γ* is a direct stimulator of PBMC thioredoxin and thioredoxin reductase (RTrx) system gene expression in human T cells [53, 54] and there is a positive feed-back circuit involving IFN-*γ* and Trx/RTrx gene expression in the regulation of intracellular reduced oxitative condition which is essential for Th1 immune response, then we can assume that the immunological response through the IFN*γ* pathway in men reduces the intracellular oxidative levels to preserve the cell oxidative balance control. In fact, male cells, as we mentioned, are incapable of maintaining an intracellular reduced oxidative condition and this would explain their greater susceptibility to diseases in which the immunological defense is prevalently Th1 type, such as tumors [47]. Similarly if we consider that the key function of IL-6 is the homeostasis within the Th cell differentiation in Treg or Th17 cells [33, 34], it is clear why women are more susceptible to diseases characterized by a lack of regulatory cell functionality such as autoimmune diseases [33, 55].

## Figures and Tables

**Table 1a:** (a) Whole blood and PBMC supernatants

Healthy subjects					
Men					
	Basic condition		PHA		LPS
pg/mL	mean^wb^ ± SD	mean^s^ ± SD	mean^wb^ ± SD	mean^s^ ± SD	mean^wb^ ± SD

IL10	5 ± 13	26 ± 40	38 ± 43	94 ± 117	61 ± 96
IFN	149 ± 194	162 ± 146	1752 ± 2344	416 ± 389	1473 ± 2408
IL6	133 ± 376	391 ± 496	236 ± 303	1386 ± 2138	573 ± 715
IL2	256 ± 182	64 ± 78	249 ± 233	290 ± 197	
IL4	15 ± 32	7 ± 10	22 ± 29	22 ± 24	

Women					
	Basic condition		PHA		LPS
pg/mL	mean^wb^ ± SD	mean^s^ ± SD	mean^wb^ ± SD	mean^s^ ± SD	mean^wb^ ± SD

IL10	26 ± 46	155 ± 181	7 ± 9	143 ± 155	73 ± 150
IFN	70 ± 110	173 ± 74	1300 ± 2294	1071 ± 1102	1603 ± 4603
IL6	35 ± 68	243 ± 239	104 ± 156	1921 ± 1654	317 ± 323
IL2	154 ± 140	239 ± 247	222 ± 193	573 ± 438	
IL4	29 ± 48	24 ± 37	77 ± 116	10 ± 5	

**Table 1b:** (b) Blood serum

*U/mL	Healthy subjects		Colorectal cancer patients		Adenoma patients	
**ng/mL	Men	Women	Men	Women	Men	Women
pg/mL	mean ± SD	mean ± SD	mean ± SD	mean ± SD	mean ± SD	mean ± SD

sIl2R*	233 ± 104	258 ± 191	520 ± 306	558 ± 240	237 ± 23	258 ± 52
SIl6R**	49 ± 38	64 ± 45	115 ± 62	139 ± 73	178 ± 47	181 ± 37
IL2	37 ± 29	68 ± 95	15 ± 36	17 ± 60	19 ± 3	86 ± 157
IFN	57 ± 121	67 ± 84	160 ± 193	146 ± 215	124 ± 89	68 ± 50
IL4	11 ± 11	11 ± 9	160 ± 244	141 ± 214	10 ± 4	21 ± 7
IL6	4 ± 11	5 ± 15	178 ± 783	68 ± 110	0.1 ± 0.2	8 ± 10
IL10	3 ± 5	3 ± 9	20 ± 25	52 ± 114	7 ± 5	6 ± 4
TNF	3 ± 9	3 ± 8	13 ± 25	11 ± 24	37 ± 4	131 ± 172
IL1	161 ± 215	171 ± 250	297 ± 300	343 ± 408	66 ± 30	77 ± 27

## References

[1] Trusheim MR, Berndt ER, Douglas FL (2007). Stratified medicine: strategic and economic implications of combining drugs and clinical biomarkers. *Nature Reviews Drug Discovery*.

[2] Dezso Z, Nikolsky Y, Nikolskaya T (2009). Identifying disease-specific genes based on their topological significance in protein networks. *BMC Systems Biology*.

[3] Piruzian E, Bruskin S, Ishkin A (2010). Integrated network analysis of transcriptomic and proteomic data in psoriasis. *BMC Systems Biology*.

[4] Martin KJ, Fournier MV, Veer Reddy GP, Pardee AB (2010). A need for basic research on fluid-based early detection biomarkers. *Cancer Research*.

[5] Presson AP, Sobel EM, Papp JC (2008). Integrated weighted gene co-expression network analysis with an application to chronic fatigue syndrome. *BMC Systems Biology*.

[6] Grossman C (1989). Possible underlying mechanisms of sexual dimorphism in the immune response, fact and hypothesis. *Journal of Steroid Biochemistry*.

[7] Schuurs AHWM, Verheul HAM (1990). Effects of gender and sex steroids on the immune response. *Journal of Steroid Biochemistry*.

[8] Cannon JG, Pierre BAS (1997). Gender differences in host defense mechanisms. *Journal of Psychiatric Research*.

[9] Rhodes K, Scott A, Markham RL, Monk-Jones ME (1969). Immunological sex differences. A study of patients with rheumatoid arthritis, their relatives, and controls. *Annals of the Rheumatic Diseases*.

[10] Butterworth M, McClellan B, Aklansmith M (1967). Influence of sex on immunoglobulin levels. *Nature*.

[11] Terres G, Morrison SL, Habicht GS (1968). A quantitative difference in the immune response between male and female mice. *Proceedings of the Society for Experimental Biology and Medicine*.

[12] Morell V (1995). Zeroing in on how hormones affect the immune system. *Science*.

[13] Homo-Delarche F, Fitzpatrick F, Christeff N, Nunez EA, Bach JF, Dardenne M (1991). Sex steroids, glucocorticoids, stress and autoimmunity. *Journal of Steroid Biochemistry and Molecular Biology*.

[14] Grossman CJ (1984). Regulation of the immune system by sex steroids. *Endocrine Reviews*.

[15] Paavonen T, Andersson LC, Adlercreutz H (1981). Sex hormone regulation of in vitro immune response. Estradiol enhances human B cell maturation via inhibition of suppressor T cells in pokeweed mitogen-stimulated cultures. *Journal of Experimental Medicine*.

[16] Schröder J, Kahlke V, Staubach KH, Zabel P, Stüber F (1998). Gender differences in human sepsis. *Archives of Surgery*.

[17] Weinstein Y, Ran S, Segal S (1984). Sex-associated differences in the regulation of immune responses controlled by the MHC of the mouse. *Journal of Immunology*.

[18] Holmdahl R, Jansson L (1988). Estrogen-induced suppression of collagen arthritis. III. Adult thymectomy does not affect the course of arthritis or the estrogen-mediated suppression of T-cell immunity. *Brain Behavior and Immunity*.

[19] Czirjak L, Bokk A, Csontos G, Lorincz G, Szegedi G (1989). Clinical findings in 61 patients with progressive systemic sclerosis. *Acta Dermato-Venereologica*.

[20] Steen VD, Medsger TA (1990). Epidemiology an natural history of systemic sclerosis. *Rheumatic Disease Clinics of North America*.

[21] Wilder RL, Elenkov IJ (1999). Hormonal regulation of tumor necrosis factor-*α*, interleukin-12 and interleukin-10 production by activated macrophages. A disease-modifying mechanism in rheumatoid arthritis and systemic lupus erythematosus?. *Annals of the New York Academy of Sciences*.

[22] Marzi M, Vigano A, Trabattoni D (1996). Characterization of type 1 and type 2 cytokine production profile in physiologic and pathologic human pregnancy. *Clinical and Experimental Immunology*.

[23] Wegmann TG, Lin H, Guilbert L, Mosmann TR (1993). Bidirectional cytokine interactions in the maternal-fetal relationship: is successful pregnancy a TH2 phenomenon?. *Immunology Today*.

[24] Raghupathy R (1997). Th1-type immunity is incompatible with successful pregnancy. *Immunology Today*.

[25] Piccinni MP, Giudizi MG, Biagiotti R (1995). Progesterone favors the development of human T helper cells producing Th2- type cytokines and promotes both IL-4 production and membrane CD30 expression in established Th1 cell clones. *Journal of Immunology*.

[26] Martin MC, Taylor RN, Kitzmiller JL, Greenspan FS, Baxter JD (1994). The endocrinology of pregnancy. *Basic and Clinical Endocrinology*.

[27] Suzuki T, Suzuki N, Daynes RA, Engleman EG (1991). Dehydroepiandrosterone enhances IL2 production and cytotoxic effector function of human T cells. *Clinical Immunology and Immunopathology*.

[28] Gilmore W, Weiner LP, Correale J (1997). Effect of estradiol on cytokine secretion by proteolipid protein-specific T cell clones isolated from multiple sclerosis patients and normal control subjects. *Journal of Immunology*.

[29] Daynes RA, Araneo BA, Dowell TA, Huang K, Dudley D (1990). Regulation of murine lymphokine production in vivo. III. The lymphoid tissue microenvironment exerts regulatory influences over T helper cell function. *Journal of Experimental Medicine*.

[30] Araneo BA, Dowell T, Diegel M, Daynes RA (1991). Dihydrotestosterone exerts a deprssive influence on the production of interleukin-4 (IL-4), IL-5, and *γ*-interferon, but not IL-2 by activated murine T cells. *Blood*.

[31] Thomson A (1994). *The Cytokine Handbook*.

[32] Banchereau J (1995). Converging and diverging properties of human interleukin-4 and interleukin-10. *Behring Institute Mitteilungen*.

[33] Cua DJ, Kastelein RA (2006). TGF-*β*, a “double agent” in the immune pathology war. *Nature Immunology*.

[34] Bettelli E, Carrier Y, Gao W (2006). Reciprocal developmental pathways for the generation of pathogenic effector TH17 and regulatory T cells. *Nature*.

[35] Desnyder R (1995). Increased plasma concentrations of interleukin-6, soluble interleukin-6, soluble interleukin-2 and transferrin receptor in major depression. *Journal of Affective Disorders*.

[36] Marx N, Imhof A, Froehlich J (2003). Effect of rosiglitazone treatment on soluble CD40L in patients with type 2 diabetes and coronary artery disease. *Circulation*.

[37] Elsasser-Beile U, Von Kleist S, Gallati H (1991). Evaluation of a test system for measuring cytokine production in human whole blood cell cultures. *Journal of Immunological Methods*.

[38] Cohen SBA, Clayton J, Londei M, Feldmann M, Rickwood D, Hames BD (1995). T cells and cytokines. *Cytokines*.

[39] Doyle A, Stein M, Keshavi S, Gordon S, Rickwood D, Hames BD (1995). Assays for macrophage activation by cytokines. *Cytokines*.

[40] Bloemena E, Roos MTL, Van Heijst JLAM, Vossen JMJJ, Schellekens PTA (1989). Whole-blood lymphocyte cultures. *Journal of Immunological Methods*.

[41] Berghella AM, Pellegrini P, Piancatelli D (1994). Progression mechanisms in colon cancer: soluble interleukin-2 (IL-2) receptor; IL-2 plus anti-CD3 proliferative response and tumor stage correlations. *Cancer Immunology Immunotherapy*.

[42] Azuaje F, Devaux Y, Wagner DR (2010). Coordinated modular functionality and prognostic potential of a heart failure biomarker-driven interaction network. *BMC Systems Biology*.

[43] Fujita A, Gomes LR, Sato JR (2008). Multivariate gene expression analysis reveals functional connectivity changes between normal/tumoral prostates. *BMC Systems Biology*.

[44] Bray D (2001). Reasoning for results. *Nature*.

[45] Janes KA, Lauffenburger DA (2006). A biological approach to computational models of proteomic networks. *Current Opinion in Chemical Biology*.

[46] Randerson PF, Fry JC (1993). Why do we need multivariate analysis?. *Biological Data Analysis. A Pratical Approach*.

[47] Powrie F, Bean A, Moore KW, Remick DG (1997). Interleukin-10. *Cytokines in Health and Disease*.

[48] Du L, Bayir H, Lai Y (2004). Innate gender-based proclivity in response to cytotoxicity and programmed cell death pathway. *Journal of Biological Chemistry*.

[49] Ortona E, Margutti P, Matarrese P, Franconi F, Malorni W (2008). Redox state, cell death and autoimmune diseases: a gender perspective. *Autoimmunity Reviews*.

[50] Hansen JM, Go YM, Jones DP (2006). Nuclear and mitochondrial compartmentation of oxidative stress and redox signaling. *Annual Review of Pharmacology and Toxicology*.

[51] Song JJ, Lee YJ (2003). Differential role of glutaredoxin and thioredoxin in metabolic oxidative stress-induced activation of appptosis signal-regulating kinase 1. *Biochemical Journal*.

[52] Kim SH, Oh J, Choi JY, Jang JY, Kang MW, Lee CE (2008). Identification of human thioredoxin as a novel IFN-gamma-induced factor: mechanism of induction and its role in cytokine production. *BMC Immunology*.

[53] Kang MW, Jang JY, Choi JY (2008). Induction of IFN-*γ* gene expression by thioredoxin: positive feed-back regulation of Th1 response by thioredoxin and IFN-*γ*. *Cellular Physiology and Biochemistry*.

[54] Oukka M (2008). Th17 cells in immunity and autoimmunity. *Annals of the Rheumatic Diseases*.

[55] Korn T, Anderson AC, Bettelli E, Oukka M (2007). The dynamics of effector T cells and Foxp3+ regulatory T cells in the promotion and regulation of autoimmune encephalomyelitis. *Journal of Neuroimmunology*.

